# Severe Cannabinoid Intoxication in a Ferret (*Mustela putorius furo*) Treated With Intravenous Lipid Emulsion

**DOI:** 10.1111/vec.70044

**Published:** 2025-10-14

**Authors:** Claudia Huerta, Amanda Lamarca, W. Y. Eunice Lam, Linda G. Martin

**Affiliations:** ^1^ Veterinary Teaching Hospital Department of Veterinary Clinical Sciences Washington State University Pullman Washington USA

**Keywords:** cannabinoid, intoxication, intravenous lipid emulsion, toxicosis, ferret

## Abstract

**Objective:**

To describe the clinical signs and outcome of a ferret with cannabinoid toxicosis diagnosed with an over‐the‐counter urine tetrahydrocannabinol test and treated with intravenous lipid emulsion (ILE).

**Case Summary:**

A 1‐year‐old spayed female ferret was admitted for evaluation of abnormal mentation after being found vocalizing under a recliner chair. The physical examination revealed severe bradycardia, bradypnea, hypothermia, and stuporous to comatose mentation. Blood analyses revealed hypercapnia and severe acidemia. Treatment for presumptive traumatic brain injury was initiated. A full body lateral radiograph and computed tomography of the skull did not reveal any signs of traumatic injuries. Seventeen hours after admission, the owner reported that the ferret potentially ingested three cannabinoid containing candies. An over‐the‐counter urine test confirmed cannabinoid intoxication. Due to the severity of clinical signs and the lack of response to supportive therapy, ILE was administered 18 and 22 h after admission with the aim of enhancing decontamination. The ferret's vital signs, neurological status, and laboratory results gradually improved. Four days after admission, the ferret was bright, alert, and responsive, neurologically normal, had a normal appetite, and was discharged from the hospital. Thirty days after discharge, the owner reported that the ferret appeared to be healthy.

**New or Unique Information Provided:**

The diagnosis of cannabinoid toxicosis in a ferret was confirmed with an over‐the‐counter human urine test, and the ferret was successfully treated for severe cannabinoid toxicosis with ILE and supportive care.

**Abbreviations**
BPM: beats per minute
CBD: cannabidiol
CT: computed tomography
HR: heart rate
ILE: intravenous lipid emulsion
iSBP: indirect systolic blood pressure
RR: respiratory rate
THC: tetrahydrocannabinol
THC‐COOH: 11‐nor‐9‐carboxy‐Δ^9^‐tetrahydrocannabinol
TP: total plasma protein concentration

## 1 Introduction

Marijuana legalization is making cannabinoid products more accessible to pets [1]. There are multiple reports of cannabinoid toxicosis in dogs and cats, but only one case of cannabinoid toxicosis in a ferret has been published. In that report, the ferret was subjected to euthanasia and a diagnosis was made postmortem [2].

In pets, cannabinoid toxicosis typically occurs after ingestion of cannabinoid products, and the common clinical signs are ataxia, hypermetria, hyperesthesia, urinary incontinence, lethargy, and mydriasis [1, 3]. In severe cases, bradycardia or tachycardia, hypothermia, seizures, tremors, hyperthermia, vomiting, and hypotension can occur [3].

The diagnosis of cannabinoid toxicosis is commonly based on the clinical signs and the history of exposure to cannabinoid‐containing products [1, 3]. Over‐the‐counter tests intended for people measure 11‐nor‐9‐carboxy‐Δ^9^‐tetrahydrocannabinol (THC‐COOH) and are not sensitive enough for the diagnosis of marijuana exposure in dogs due to the low concentration of THC‐COOH in canine urine [4].

There is no specific antidote for cannabinoids [3]. Therefore, treatment of cannabinoid toxicosis is based on decontamination and supportive care [3].

This report aims to describe the clinical signs of a ferret intoxicated with cannabinoids after ingestion of cannabinoid‐containing candy, the diagnosis with an over‐the‐counter urine test after ruling out other possible etiologies for its clinical signs, and treatment with intravenous lipid emulsion (ILE) and supportive care.

## 2 Case Summary

A 1‐year‐old spayed female ferret was admitted to Washington State University Veterinary Teaching Hospital with neurological signs including seizure‐like activity, vocalization, and tremors. The owner suspected that the ferret had been crushed by a recliner chair approximately 60 min before admission.

On admission, the ferret was stuporous and in lateral recumbency; the oral mucosa was tacky, pale pink with a capillary refill time < 2 s; no evidence of bleeding, wounds or fractures were detected on inspection or palpation; body weight was 0.75 kg (0.65–0.95 kg) [5]; heart rate (HR) was 128 beats per minute (BPM) (200–400 BPM) [5]; respiratory rate (RR) was 6 BPM (33–36 BPM) [5]; and rectal temperature was 35.9°C (97.6°F) (37.8°C–40.0°C [100°F–104°F]) [5]. Indirect systolic blood pressure (iSBP) measured on the left metatarsus via Doppler ultrasonic flow probe was 110 mmHg (95–155 mmHg) [5]. Blood collected from the right jugular vein revealed a PCV of 50% (40%–70%) [5], blood glucose concentration of 14.9 mmol/L (269 mg/dL) (6.66–7.99 mmol/L [54–153 mg/dL]) [6], lactate concentration of 0.8 mmol/L (0.3–1.5 mmol/L) [5], estimated BUN of 5–15 mg/dL (5–15 mg/dL) [5]. The concentration of total plasma protein (TP) was not recorded.

An ECG did not reveal any arrhythmia. Point‐of‐care ultrasonography of the abdomen and thorax was unremarkable. Neurological examination revealed no pupillary light reflex, menace, corneal reflex, or palpebral reflexes bilaterally; a resting strabismus with ventromedial rotation OS; anisocoria with mydriasis OS; minimal jaw tone; absent facial sensation, nasocortical response, gag reflex, and tongue movement; no proprioceptive reflexes; minimal withdraw reflexes of all limbs; and reduced anal tone. These findings were suggestive of diffuse intracranial disease or abnormalities.

Initial treatment included flow‐by oxygen administration, covering the ferret with a forced‐air heating blanket,^1^ mannitol^2^ administration (two 0.5 g/kg doses over 15 min) via a 26‐Ga catheter placed in the left cephalic vein to address suspected intracranial hypertension. Then, a bolus (5 mL/kg iv) of a balanced electrolyte solution^3^ was administered. Since mentation did not improve and iSBP and rectal temperature dropped to 70 mmHg and 33.4°C, respectively, hypertonic saline^4^ (3 mL/kg iv over 5 min) was administered and iv administration of a balanced electrolyte solution was initiated at 100 mL/kg/day (maintenance rate, 75–100 mL/kg/day) [5]. Twenty minutes after the administration of hypertonic saline, the iSBP increased to 110 mmHg, HR and RR were 145 and 8 BPM, respectively, and the ferret became minimally responsive. Thirty minutes later, iSBP dropped to 80 mmHg and the ferret's mentation became comatose. Due to the severity of clinical signs and unresponsiveness to medical management, dexamethasone sodium phosphate^5^ (0.1 mg/kg iv) was administered with the aim of minimizing cerebral inflammation.

Due to concerns about musculoskeletal injuries and brain edema caused by the suspected trauma, the ferret was secured with adhesive tape to a hard board, which was tilted 30° to keep the head elevated and housed in a temperature and oxygen‐controlled chamber^6^ (26.7°C, 40% oxygen). Analysis of a venous blood sample after mannitol and hypertonic saline revealed a decreased pH (7.065 [7.274–7.415]) [7], increased PvCO_2_ (126.7 mmHg [35.2–59.1 mmHg]) [7], decreased oxygen saturation (45.6% [55.3%–93.3%]) [7], increased bicarbonate concentration (36.3 mmol/L [21.3–30 mmol/L]) [7], increased TCO_2_ (37.6 mmol/L [22.6–31.6 mmol/L]) [7], mildly reduced ionized calcium concentration (0.97 mmol/L [1.14–1.35 mmol/L]) [7], a creatinine of < 26.53 µmol/L (< 0.3 mg/dL) (8.8–106 µmol/L)[0.1–1.2 mg/dL]) [6], and decreased PCV (32% [40%–70%]) and normal potassium concentration (4.3 mmol/L [3.9–5.1 mmol/L]) [7]. The TP was not recorded. Full body lateral radiography did not reveal any fractures, luxations, or obvious soft tissue injuries.

Three hours after admission, the ferret's HR dropped to 45 BPM while it was still comatose with anisocoria and no jaw tone. A third dose of mannitol (0.5 g/kg iv over 15 min) was administered and the HR increased to 130 BPM.

Twelve hours after admission, the ferret remained stuporous and bradypneic, but the strabismus had resolved and pupillary light reflex, mild facial sensation, palpebral reflex, anal tone, and deep pain on the left pelvic limb were present. Since iSBP gradually declined, a total of five iv boluses (5 mL/kg each) of a balanced electrolyte solution were administered over the initial 12 h following presentation, and the iSBP improved to 75 mmHg. Fluid therapy was increased to 190 mL/kg/day. At that time, the urine specific gravity was 1.041 (1.026–1.060) [7], blood glucose concentration was 18.44 mmol/L (332 mg/dL), PCV 42%, and TP 60 g/L (6 g/dL) (43–60 g/L) [4.3–6 g/dL]) [6]. Approximately 14 h after admission an iv fluid bolus (10 mL/kg of a balanced electrolyte solution) was administered, and the blood pressure increased from 50 to 82 mmHg, and the fluid rate was increased to 250 mL/kg/day (approximately 2.5 times the maintenance iv fluid rate), due to the responsiveness of the blood pressure to the fluid bolus. After this last fluid bolus, the acid‐base status improved (pH, 7.277; PvCO_2_, 69.3 mmHg; TCO_2_, 32.2 mmol/L; HCO_3_, 32.4 mmol/L) and PCV decreased (27%) while TP did not change. Due to the lack of clinical improvement and the suspected history of traumatic brain injury, a second dose of dexamethasone sodium phosphate (0.1 mg/kg iv) was administered, and plain and contrast‐enhanced computed tomography (CT) was performed without any sedation. No evidence of any structural abnormalities was found.

Following the CT, the owner reported that three cannabinoid candies^7^ (10 mg of cannabinoid and 10 mg of cannabinol per candy) were missing and that another ferret in the household was lethargic but had a good appetite. An over‐the‐counter urine test^8^ designed for human use revealed cannabinoids in the patient ferret's urine. Due to the ferret's abnormal mentation and the possibility of traumatic brain injury, induction of emesis and administration of activated charcoal were not performed. Due to the lack of improvement with supportive care and concerns for further complications secondary to stuporous mentation including aspiration pneumonia, lipid emulsion^9^ was administered iv as recommended for dogs and cats (1.5 mL/kg bolus over 15 min, followed by 0.25 mL/kg/min for 1 h [total, 15.5 mL/kg]), which did not change the mental status or resolve the bradycardia or bradypnea. Lipid emulsion administration was then repeated 4 h later, when the ferret's serum was no longer lipemic. Following the second administration of lipid emulsion, the ferret became significantly more responsive, moving voluntarily around the kennel with stronger neurological reflexes present (pupillary light reflex, palpebral reflex, gag, and proprioception).

Forty‐eight hours after admission, the ferret was quiet and responsive, had normal temperature and RR, but it was still bradycardic. Oxygen therapy was reduced to FiO_2_ 25%. Since the ferret appeared to be well hydrated, fluid therapy was decreased. Since a gag reflex was present, water and food were offered, and the ferret voluntarily ate soft food and appeared to have a normal appetite.

Seventy‐two hours after admission, the ferret was bright, alert, and responsive. Its vital signs continued to improve, and fluid therapy and oxygen therapy were discontinued. The ferret was urinating and defecating normally, appeared to be well hydrated, and had a normal appetite. At the time of discharge, 96 h after admission, its mentation was normal, it was active and playful, and it was fully ambulatory. Thirty days after discharge, the owner reported that the ferret appeared to be healthy and was doing well at home.

## 3 Discussion

The present report describes a case of severe cannabinoid toxicosis in a ferret that was diagnosed with an over‐the‐counter human urine test and was successfully treated with ILE administration and supportive care. This case illustrates that ingestion of cannabinoid candy can produce severe neurologic signs in ferrets, such as markedly decreased mentation, absent gag reflex, anisocoria, bradycardia, hypothermia, hypotension, and hypercapnia.

The severe hypercapnia present in this patient warranted mechanical ventilation or high‐flow nasal oxygen therapy; our facility does not have these capabilities to treat patients weighing < 1 kg. Manual ventilation could be considered; however, in this case, it was declined by the owners. Due to the inability to ventilate the patient, it was decided to provide oxygen therapy via oxygen cage at 40% FiO_2_ to increase PAO_2_. Hypoxemia due to hypoventilation can be improved with supplemental oxygen by increasing PAO_2_ even if the hypoventilation remains uncorrected [8]. The PvCO_2_ improved significantly within 14 h, decreasing from 126.7 to 69.3 mmHg.

Due to the severity of clinical signs and poor response to initial medical management, including several doses of hyperosmolar therapy to reduce suspected intracranial edema, and the owners' consideration of euthanasia based on the lack of clinical improvement, dexamethasone sodium phosphate was administered with the aim of minimizing intracranial edema. However, a controversial approach, recent human studies suggest a benefit from the use of corticosteroids in some cases of acute brain injury due to their neuroprotective effects [9, 10, 11]. These effects are based on affecting downstream apoptotic pathways and decreasing neuronal cell death, reducing tumor necrosis factor alpha production, limiting the inflammatory‐mediated response, and acting on prosurvival signals [10]. Also, dexamethasone exerts neuroprotection via activation of phosphorylated Akt, similar to the effects seen in hypothermia treatment [10]. Adverse effects of dexamethasone, such as gastrointestinal ulceration and polyuria, could have further compromised the ferret; however, there is no published evidence of these adverse effects in ferrets. This ferret's clinical improvement was obvious only after the intralipid administration, which occurred several hours after corticosteroid administration. Thus, the effect of corticosteroids in this case was unclear, and they most likely did not impact the ferret's clinical improvement.

Although normal in this case, electrolyte disturbance should be considered as a potential cause of the neurological signs or a worsening factor, especially in the event of hypokalemia and hypomagnesemia. Clinical signs of hypokalemia are rare in other species and can include neuromuscular weakness, ventral flection of the neck, stiff hindlimbs, wide‐based stance, and hypermetria of the forelimbs [12]. Severe cases can result in respiratory arrest from neuromuscular weakness. Severe hypomagnesemia can also lead to muscle weakness, neurological signs, abnormal behavior, and sudden death [12]. Magnesium concentrations were not measured in this ferret becuase the blood volume required would have exceeded the total blood volume extraction recommended for this species within 24 h [13]. Empirical supplementations could be considered in cases where prolonged anorexia was present or anticipated.

The LD_50_ for cannabinoids has not been established in ferrets, dogs, or cats [3]. In one study, the LD_50_ of cannabinoids administered intragastrically and by inhalation to rats was found to be 36–40 mg/kg [14]. In dogs, the minimal toxic or lethal dose of delta‐9‐THC is 3 g/kg when taken orally, which is 1000 times greater than the dose that causes behavioral disorders [3]. In the present report, the maximum ingested dose could have been 30 mg (40 mg/kg) of each one of the two compounds present in the candy (THC and cannabidiol [CBD]). However, since another ferret in the household was also affected, the dose ingested by the ferret in this report was likely lower than 40 mg/kg of THC and CBD. In dogs, the onset of clinical signs of marijuana toxicosis typically occurs within 30–90 min of exposure and can last up to 96 h [3]. In this ferret, it was not possible to determine how soon the clinical signs appeared after exposure because the owner did not know when the ferret ingested the cannabinoid‐containing candy or when the ferret started showing clinical signs of intoxication.

It is unclear what the cause of the ferret's high fluid requirements is, but it was likely multifactorial. It is possible that fluid losses (urine output) were underestimated since a urinary catheter was not placed and the urine output was not measured. An underestimation of the degree of dehydration is also possible since the patient's clinical signs may have started hours before presentation. Repeated doses of mannitol could have contributed to increased urine output, which clinicians were likely not matching. Also, the lack of oral water intake because of the ferret's altered mental state has to be considered. Other potential causes could include CBD‐induced hypotension as seen in other species [15], or CBD caused endothelium‐dependent vasorelaxation of the mesenteric arteries via CB1 activation, which has been reported in people [16].

In dogs, over‐the‐counter drug tests for marijuana are known not to be adequately sensitive because of the relatively low concentrations of THC‐COOH in dog urine, which is the cannabinoid metabolite detected by over‐the‐counter tests [4]. Other causes of false negative results include sampling urine too soon after cannabinoid exposure; sample handling and storage problems, since THC binds to rubber stoppers and glass; consumption of synthetic cannabinoids; and overhydration [17]. In this ferret, the positive urine results may be explained by a higher elimination rate of THC‐COOH in urine relative to dogs. Considering that this ferret weighed < 1 kg, it is also possible that the ingested dose per kg of body weight was much higher in this ferret than what is typically observed in dogs. Although the possibility that the urine result could have been a false positive has to be considered, the history, clinical signs, and response to treatment strongly suggest that this was indeed a case of cannabinoid toxicosis. An example of a possible cause for a false positive result would be the treatment with nonsteroidal anti‐inflammatory drugs [18]; however, this ferret was not treated with such medications. Other modalities of cannabinoid detection and quantification exist, such as liquid chromatography–tandem mass spectrometry [19] or gas chromatography [20], which can be considered in cases where urine testing is negative, but a high suspicion remains.

Lack of evidence of traumatic injuries on radiographs and CT did not support the hypothesis of trauma. The CT was not performed until approximately 14 h after admission since this modality is only available during routine hours in our hospital. Later, the history of missing cannabinoid candy and the positive urine test supported the diagnosis of cannabinoid intoxication.

In veterinary medicine, only acute cannabinoid toxicosis has been described, and the treatment for this condition is similar to what is recommended for people. Emesis induction and activated charcoal administration can be considered to prevent absorption of ingested cannabinoids still present in the gastrointestinal tract [21]. These approaches were not used for this ferret because the ferret's neurological deficits were a contraindication for emesis induction and oral administration of activated charcoal. Treatment with extracorporeal therapy should be considered in extreme intoxications such as this case [22]. However, due to the small size of this patient (0.75 kg), this treatment option would be extremely challenging, if not impossible.

Cannabinoids are highly lipophilic, are distributed in adipose tissue, liver, lungs, and spleen after absorption from the gastrointestinal tract, and are slowly redistributed into the plasma before excretion in the feces and urine [23]. ILE has been described as an effective treatment for toxicosis caused by lipophilic substances such as calcium channel blockers, bromethalin, avermectin parasiticides, baclofen, bupropion, loperamide, permethrin in cats, and sertraline [3]. It has been hypothesized that an expanded intravascular lipid phase acts to sequester lipophilic toxin within it, thus reducing the effect site concentration and toxicosis until the compound is metabolized and excreted [24]. In the ferret described in this report, ILE was administered as soon as the diagnosis of cannabinoid toxicosis was confirmed by the urine test. In this case, no clinical improvements were seen until after the second ILE treatment (total dose 31 mL/kg). Conversely, in the only reported ferret treated with ILE, a much lower dose (total dose 9 mL/kg) was effective for olanzapine toxicosis [25]. The large volume of lipid emulsion required in the present ferret may be explained by differences between the toxic compounds (olanzapine vs. cannabinoids) and/or the ingestion of a very high dose of cannabinoids by this ferret.

Potential adverse effects of ILE administration in small animals can include pancreatitis secondary to persistent lipemia, hypersensitivity reactions, fat overload syndrome, and lipid emboli, as well as sepsis [26]. Lipemia was subjectively diagnosed in the ferret reported here immediately after administration of the first dose of ILE; however, 4 h later, gross evidence of lipemia was no longer observed. Consequently, treatment with ILE in this ferret did not cause obvious complications.

In conclusion, severe cannabinoid toxicosis in this ferret was successfully managed with supportive care and ILE. The history of possible trauma delayed the diagnosis of cannabinoid toxicosis. Considering that cannabinoid‐containing products are now widely available in North America, it is essential to consider cannabinoid toxicosis as a differential in pets with signs of diffuse dysfunction of the central nervous system. More studies are needed to provide essential information that can help diagnose cannabinoid toxicosis in ferrets and determine whether over‐the‐counter urine tests can be routinely used to detect THC‐COOH concentrations in ferret urine.

## Author Contributions

**Claudia Huerta**: project administration, writing – original draft, writing – review and editing. **Amanda Lamarca**: writing – original draft, writing – review and editing. **W. Y. Eunice Lam**: writing – original draft, writing – review and editing. **Linda G. Martin**: writing – review and editing

## Conflicts of Interest

The authors declare no conflicts of interest.

## Endnotes

1 Bair Hugger Model 505, 3M HealthCare, St. Paul, MN.

2 Mannitol 20%, VETONE, Boise, ID.

3 Vetivex, Dechra Veterinary Products, Overland Park, KS.

4 Hypertonic saline 7.2%, VETONE, Boise, ID.

5 Dexamethasone‐SP, VETONE, Boise, ID.

6 Intensive Care Unit, Snyder Mfg. Co., Centennial, CO.

7 Cannabis‐Infused Pomegranate Gummies, Wyld, Clackamas, OR.

8 Easy@Home Single Panel Marijuana/THC Urine Drug Test, Burr Ridge, IL.

9 Intralipid 20%, Baxter Healthcare Corporation, Deerfield, IL.
